# Production of genetically and developmentally modified seaweeds: exploiting the potential of artificial selection techniques

**DOI:** 10.3389/fpls.2015.00127

**Published:** 2015-03-17

**Authors:** Bénédicte Charrier, Elodie Rolland, Vishal Gupta, C. R. K. Reddy

**Affiliations:** ^1^Centre National de la Recherche Scientifique, Sorbonne Université, UPMC Univ Paris 06, UMR 8227, Integrative Biology of Marine Models, Station Biologique de RoscoffRoscoff, France; ^2^Seaweed Biology and Cultivation Group, Division of Marine Biotechnology and Ecology, CSIR-Central Salt and Marine Chemicals Research InstituteBhavnagar, India

**Keywords:** seaweed, somatic hybridization, mutagenesis, varietal improvement, genetic manipulation

## Abstract

Plant feedstock with specific, modified developmental features has been a quest for centuries. Since the development and spread of agriculture, there has been a desire for plants producing disproportionate—or more abundant and more nutritional—biomass that meet human needs better than their native counterparts. Seaweed aquaculture, targeted for human consumption and the production of various raw materials, is a rapidly expanding field and its stakeholders have increasing vested interest for cost-effective and lucrative seaweed cultivation processes. Thus, scientific research on seaweed development is particularly timely: the potential for expansion of seaweed cultivation depends on the sector's capacity to produce seaweeds with modified morphological features (e.g., thicker blades), higher growth rates or delayed (or even no) fertility. Here, we review the various technical approaches used to modify development in macroalgae, which have attracted little attention from developmental biologists to date. Because seaweed (or marine macroalgae) anatomy is much less complex than that of land plants and because seaweeds belong to three different eukaryotic phyla, the mechanisms controlling their morphogenesis are key to understanding their development. Here, we present efficient sources of developmentally and genetically modified seaweeds—somatic variants, artificial hybrids and mutants—as well as the future potential of these techniques.

## Societal importance of seaweeds

Marine macroalgae (seaweeds) are one of the renewable resources in marine ecosystems and carry out several key ecosystem functions that contribute to the productivity of the oceans. Seaweeds are traditionally consumed as human food in several Asian countries, where they are cultivated on a large scale. A wide range of seaweeds belonging to different genera have been known as a food source since prehistoric times (Mouritsen et al., 2013). In most cases, the entire thallus of the seaweed is consumed (e.g., species of *Porphyr*a and *Enteromorph*a), although, in some cases, only certain parts are edible (e.g., in *Caulerpa lentillifera*, only the ramuli are edible). In larger seaweeds, such as *Laminaria digitata* and *Undaria pinnatifida*, the stipe is used for preparing local dishes, such as soup. *Chondrus crispus* and certain species of *Gracilaria* are consumed in salad and in these cases only tender tips are used. Seaweeds are good sources of high-quality digestible proteins with a balanced amino acid composition, polyunsaturated fatty acids including omega-3 and omega-6 and important vitamins and minerals, have a caloric content similar to land crops and are high in fiber (MacArtain et al., 2007).

According to recent statistics published by the FAO (2014), seaweed production has increased from less than 4 million wet metric tons (t) in 1980 to almost 20 million wet t in 2012, with more than 50% of total production is used for direct human consumption. The indirect products derived from seaweeds, polysaccharides in particular, have unique applications in processed as well as functional foods, pet foods, feed, fertilizers, cosmetics, and medicines. Furthermore, recent bioprocessing techniques have also unequivocally demonstrated that seaweeds are potential feedstock for production of biofuels and commodity products (Baghel et al., 2015).

The major seaweed species that are currently farmed include *Kappaphycus alvarezii* and *Eucheuma* spp. both known as “cottonii” (>8 million wet t) followed by *Saccharina japonica* (formerly *Laminaria japonica*)—known as “kombu” (5.6 million wet t), *Gracilaria* spp. (~3 million wet t), *U. pinnatifida*—known as “wakame” (2 million wet t) and *Porphyra* spp.—known as “nori” (1.8 million wet t) (Buchholz et al., 2012). The use of *Kappaphycus* enhances the texture of fish cutlets and pork patties and further increases the nutritional value of foodstuffs by providing minerals (Senthil et al., 2005; Jeon and Choi, 2012). Other species such as *Palmaria*, *Chondrus*, and *Ulva* are produced on a lower scale (FAO, 2014). The global utilization of various seaweed species is described in Supplementary Table 1. Seaweed cultivation is now perceived as an excellent alternative source of revenue for coastal fishermen, particularly in light of its advantages, including a shorter production cycle, low capital outlay and relatively simple farming techniques. There have been some biotechnological advances to improve economically important seaweeds. The present article briefly reviews the various research efforts that have been made to produce morphological variants of economically important seaweeds and study the genetic basis behind the changes in their morphology.

## Genetic manipulation and improvement in seaweeds

### Somaclonal variants

Cellular biotechnology in seaweeds was initiated in the 1980s and lags far behind that of terrestrial plants.

**Figure 1:**
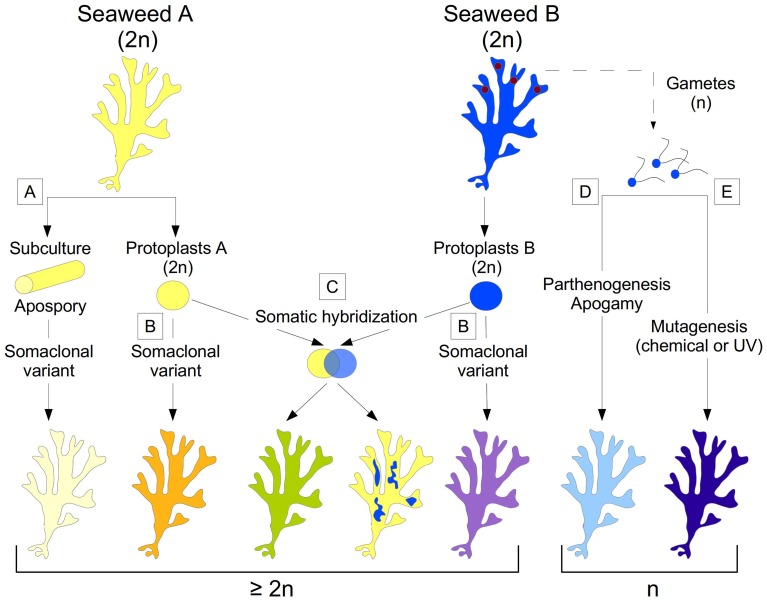
**Summary of the different technological approaches for generating morphologically modified macroalgae**. Diploid sporophytes are shown as the initial material. Change in color states indicates change in morphology. The more the color is different from the original plant, the more distinct the phenotype is. Dashed lines show cases that involve an alternation of generations (e.g., from sporophyte to gametophyte). **(A)** Sub-culture of seaweed fragments and apospory; **(B)** Protoplast preparation. **(C)** Somatic hybridization producing thalli with either novel characters (green) or chimeric or mixed parental characters (yellow and blue patches). **(D)** Parthenogenesis giving rise to morphological variants. **(E)** Chemical or UV-mediated mutagenesis on gametes. Ploidy often increases when **(A–C)** are employed.

In macroalgae, the development of *in vitro* culture systems facilitates mass proliferation of biomass all year round for the production of valuable compounds under controlled conditions. Culture systems are also a source of novel genetic variants with useful traits arising from somaclonal variation (Figure 1A). The techniques for somatic embryogenesis or dedifferentiation of somatic cells often induce morphological variants in seaweeds. Such morphological and developmental variation has advantages for genetic improvement programs and also can be used as an efficient means of germplasm storage and selection. The new variation can be transient, reversible or permanent. Temporary changes are mainly due to either epigenetic or physiological changes, which can be reversible, even after being heritable for a few generations (Kaeppler et al., 2000). The underlying molecular mechanisms involved in permanent somaclonal variants are rarely investigated and little understood (Larkin and Scowcroft, 1981). Nevertheless, most morphological variation observed during *in vitro* culture of seaweeds is transient and is not passed on to progeny. There are many examples where same genotype can produce different morphological phenotypes. For example: *in vitro* tissue culture of kelp (order Laminariales) sporophytes have a frequent developmental pattern in which outgrowths of aposporous gametophyte-like filaments with differentiated fertile branches can give rise directly to sporophytes (Ar Gall et al., 1996). Similarly, meristem cultures of *Laminaria* regenerate into one of three different body types: (1) uniseriate filaments; (2) thalloid-like structures; (3) dark green, compact calli. Analogously, early development of embryonic germlings from 22 species of Fucaceae can show up to six different developmental types.

**Figure 2:**
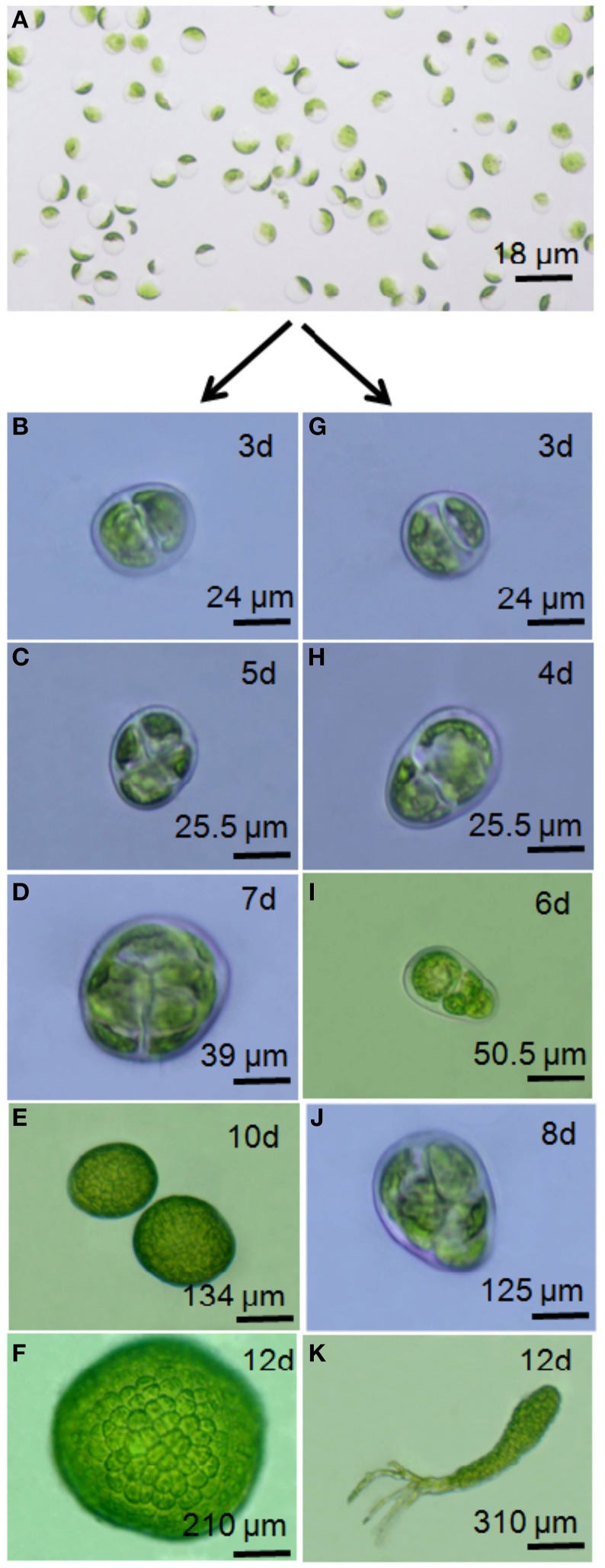
**Illustration of developmental variation in marine macroalgae upon protoplast generation**. Developmental variants among protoplast-derived germlings of *Ulva reticulata* Forsskål C (Source: Gupta et al., 2012). *U. reticulata* protoplasts **(A)** regenerate either into a callus **(D–F)** due partly to symmetrical cell divisions **(B,C)** or into an organism displaying a developmental pattern similar to that of native organisms **(G–K)**. Note the asymmetrical division taking place in **(G)**, better seen in **(H)** after cell enlargement.

Plant protoplasts are amenable to *in vitro* genetic manipulation techniques for developing genetically improved strains of agronomic crops. There have been numerous studies on the isolation and regeneration of protoplasts from a wide variety of seaweed body types ranging from simple leafy thalli to complex, cylindrical, branched thalli (see review Reddy et al., 2008). Unlike higher plants, seaweed protoplasts regenerate and differentiate directly into a complete thallus without any addition of phytohormones in the culture medium. Protoplasts from green seaweeds nevertheless have different types of morphogenetic patterns (Figure 1B) and give rise to several phenotypically variable morphotypes, such as free-living sporangia, microthalli, or saccate (or spherical), tubular (or spindle), irregular, or frondose thalli with various life spans (Reddy et al., 1989; Huang et al., 1996; Chen, 1998; Krishna Kumar et al., 1999; Chen and Shih, 2000; Rusig and Cosson, 2001). In the red alga *Porphyra*, three different types of protoplast regeneration patterns have been described: (1) callus form, (2) filamentous form and (3) conchocelis form (Polne-Fuller et al., 1984; Fujita and Migita, 1985; Waaland et al., 1990; Dipakkore et al., 2005). Callus-like outgrowth (i.e., an unorganized cellular mass) has been reported from protoplast culture of various brown macroalgal species. Similarly, two distinct patterns of development have been observed for protoplast-based development of *Gracilaria gracilis*, giving rise to plants that differ in appearance and life span. Regenerated plants either resemble parental plants, with slender, branched thalli, or remain small with thick, unbranched thalli, many of which die. Although the underlying mechanisms have been poorly studied, this variation in developmental morphological abnormalities has been attributed to a variety of factors, including the type of donor tissue from which protoplasts are prepared and the culture conditions employed for regeneration. For example, protoplasts isolated from the vegetative thalli of *Monostroma latissimum* regenerate into normal thalli, whereas protoplasts isolated from the holdfast develop into filaments (Chen, 1998). Protoplasts from *Ulva fasciata* develop into microthalli when cultured in high density (Chen and Shih, 2000). For brown seaweeds in the order Laminariales, protoplast regeneration into normal sporophytes can occur via different developmental processes, such as direct regeneration into plantlets (in *L. digitata*; Benet et al., 1997, *U. pinnatifida*; Matsumura et al., 2001 and *L. japonica*; Sawabe and Ezura, 1996; Sawabe et al., 1997; Matsumura et al., 2000), or indirect regeneration, depending on water temperature in some species. Indirect regeneration occurs either after de-differentiation of the tissue into a filament (in *U. pinnatifida*; Matsumura et al., 2001, *L. saccharina*; Benet et al., 1997) or after the development of callus-like masses [*U. pinnatifida*; (Matsumura et al., 2001) and *L. japonica*; Matsumura et al., 2000]. Gupta et al. (2012) provide the first report of epigenetic regulation of morphology in protoplast-derived germlings, with DNA methylation acting as an underlying molecular mechanism in protoplast-derived germlings in *Ulva* r*eticulata* (Figure 2). Axenic culture conditions may also cause the development of abnormal thalli. Most studies have shown that seaweed-associated microflora produce certain morphogenetic substances that, in turn, lead to normal thallus structure (Matsuo et al., 2005; Spoerner et al., 2012). However, most commercial seaweed cultivation is currently based on simple vegetative propagation due to economic and farming advantages. The *in vitro* culture techniques currently being developed for seaweeds can create new genetic variants or promote clonal propagation in photobioreactors for high end applications.

### Inducing morphological variation through somatic hybridization

In addition to morphological variation induced by *in vitro* culture, hybridization is an important process that combines phylogenetically distinct genetic lineages and results in morphotypes that are either intermediate to the parental species or completely novel (Figure 1C). In seaweeds, species are not always accurately defined and are constantly being delineated through molecular genotyping or sequencing (Maggs et al., 2007). Through the crossing of two genetically distinct species, hybridization can bring together divergent genetic lineages (Hodge et al., 2010). The resulting hybrid either combines parental phenotypes or results in a new phenotype quite distinct from the parental type. In addition to natural hybridization events that lead to speciation, somatic hybridization via protoplast fusion offers great promise for achieving wide crosses between species that are difficult or impossible to hybridize conventionally (Davey et al., 2005). Fusing protoplasts of different origins harnesses natural genetic diversity and provides novel genetic combinations resulting in the potential improvement of various functional traits. However, protoplast fusion leads to either heterokaryon (fusion of nuclei of different species) or homokaryon (fusion of nuclei of same species) recombination. In both cases, the increase in ploidy itself can improve agronomic traits. Most studies use color differences to distinguish heterokaryons from homokaryons and from unfused parental protoplasts. Somatic hybridization has shown unprecedented success in terrestrial plants but few attempts have been made in seaweeds (Reddy et al., 2008). Furthermore, a detailed description of cell division and developmental stages is still lacking in almost all the protoplast fusion studies published to date. Below, we provide a brief overview of the regeneration of fusion products so far reported from different seaweed species.

#### Intrageneric hybridization

The first report of protoplast fusion and fusion product regeneration between two color morphs of *Porphyra* was that of Fujita and Migita (1987) using the polyethylene glycol (PEG) method. Protoplast fusion from two different color types (green and reddish purple) in *Porphyra yezoensis* UEDA gave rise to callus-like masses that were composed of greenish and reddish-purple cells of various diameters. The young thalli grown from these cell masses attained a length of about 60 cm and were composed of microscopically chimeral tissues (as observed in the calli), irregularly variegated with greenish and reddish purple cell groups and possibly due to the independent segregation of two chloroplast types from the fusion partners. Further studies on sexual crosses between green mutant and wild-type red *P. yezoensis* demonstrate the production of uniform wild-type color and green mutants along with mutants consisting both of wild-type patches and green mutant patches (Niwa et al., 2002). The mechanisms behind the formation of chimeric thalli from protoplast fusion clearly differ from those involved in natural crosses. Protoplast fusions between other *Porphyra* species such as *P. yezoensis* and *P. pseudolinearis* give rise to plants resembling either one or the other parent or occasionally mosaic chimeric plants with different color contours (Fujita and Saito, 1990). Similar mechanisms of independent segregation of chloroplasts have been reported in various studies on higher plants (Cocking, 1983; Davey et al., 2005). These results reveal that crossing two species by protoplast fusion or normal sexual crosses shows different developmental processes. The possible reason for the development of chimeric thalli may be chromosomal complementation from both fusion partners, whereas in natural crosses, only one set of chromosomes from each fusion partner is found in the resulting genotype.

Protoplast fusion between two *Gracilaria* species, i.e., between *G*. *tikvahiae* (green pigmented) and *G. chilensis* (red pigmented) result in bicolor, red and green chimeric plants (Cheney, 1990). The hybrids exhibit several unusual morphological features, such as branching morphology unlike either of the parental plants. Limitations in the regeneration of somatic hybrids have led some researchers to fuse newly released spores. Kapraun (1989, 1990) developed parasexual hybrids from the fusion of zoospores from *Enteromorpha* (now *Ulva*) and *Ulvaria*. Most hybrid germlings showed morphology similar to *Enteromorpha*, although a few showed multinucleate thalli with “giga” characteristics, including larger cells with greatly enlarged vacuoles (nuclei in “gigas” vegetative cells are 8–10 μm diameter compared with 5–6 μm diameters in control plants). Mizukami et al. (1995) demonstrated protoplast fusion between *P. suborbiculata* and *P. yezoensis*. The hybrids initially showed repeated cell divisions and formed a multicellular body, which, upon subsequent culturing, produced rhizoids. The germinated hybrids showed thalli morphologically different from each other as well as from the fusion partners. The thallus of *P. suborbiculata* is brownish and roundish, and possesses spike-like serrations on the margin, whereas the thallus of *P. yezoensis* is greenish and slender and has smooth margins. Hybrid thalli were usually brownish and had a roundish, long and slender shape—thus combining features of the parental thalli—and wrinkled margins.

#### Intergeneric hybridization

Subsequent studies on intergeneric protoplast fusion have been carried out between morphologically different strains of *Ulva pertusa* and *Enteromorpha prolifera* (Reddy et al., 1992). Presumptive heteroplasmic fusion products were identified based on their larger size and the presence of twin chloroplasts. Subsequent analysis on regeneration patterns of fused protoplasts showed that they were similar to normal (unfused) protoplast development. Most of the regenerated plants from fusion products had a thallus similar to either *U. pertusa* or *E. prolifera*. However, the thalli of some plants had a characteristic irregular and dentate margin, which has never been observed in the parental type.

#### Transdivisional hybridization

Kito et al. (1998) published the first report of successful transdivisional protoplast fusion between *Monostroma* and *Porphyra*. Protoplast fusion products of these two species showed different regeneration processes and characteristics. Although the fusion partners displayed distinct monostromatic and distromatic thalli, the regenerated hybrids were green with a distromatic structure. Initial heterokaryons were identified based on clearly distinguishable chloroplast colors, but were indistinguishable after 5 days of culture. Of the hybrids generated, one of the heterofusant plants grew into a multicellular body followed by the development of rhizoid-like and bud-like organs. Finally, the plant grew into a long stringy plant (1.5 m in length and 1.5 cm in width) and was named “cyojo.” Another hybrid grew a multicellular body, which, upon subsequent culturing, separated into individual cells, each one growing into a long stringy plant; this mutant was named “kattsunbo.” A third fusion product grew into a bud. This bud-like plant became a thallus (7.5 cm in length and 6.5 cm in width) and was named “nigo.”

These few examples illustrate the range of morphological alterations that can be generated by somatic hybridization in seaweeds. Furthermore, procedures have been developed to isolate seaweed protoplasts and bring them to the full thallus regeneration stage on a wide range of seaweeds including the most anatomically complex taxa such as *Laminaria*, *Undaria*, *Gracilaria*, and *Kappaphycus*—which are some of the most commercially important seaweed genera. Nevertheless, somatic hybrids have not been widely developed and have not yet produced “cultivars” for field cultivation. However, the progress made thus far with the development of homo- or hetero-karyons via somatic hybridization provides useful groundwork for continued research in the development of macroalgae with improved functional traits.

### Inducing morphological variation through parthenogenesis

Parthenogenesis can occur either through apospory (Figure 1A) or apogamy (Figure 1D) without any ploidy changes. In the former, diploid gametophytes are produced directly by sporophytic cells whereas, in the latter, haploid sporophytes produced directly from gametophytic cells. In most cases, parthenogenetic plants are identical to wild types but occasionally give rise to phenotypic variants. Parthenogenetic proliferation (asexual reproduction) has been extensively reported for several taxa of brown and green seaweeds cultured *in vitro*, but are rarely reported in red algae. Tatarenkov et al. (2005) observed dwarf morphs of *Fucus vesiculosus* along with common morphs in wild populations itself in the brackish water Baltic Sea. The dwarf morphs may have evolved from common morphs in response to the prevailing low salinity habitat. Shan et al. (2013) reported induction of larger parthenogenetic sporophytes from gametophytic clones of *U. pinnatifida*. Furthermore, the blades of parthenogenetic sporophytes were uniformly smooth and without wrinkles on either side of the mid-rib. The genetic features of parthenogenetic phenotypes have also been investigated for *Laminaria* and *Undaria*. Parthenogenetic plants with morphological variation can lead to new insights into the genetics of the relationship between ploidy level and morphology. These parthenogenetic plants can also be used as a potential resource for breeding studies for the genetic improvement of macroalgal strains of aquaculture importance.

### Mutagenesis-mediated morphological variations

Macroalgal mutants have been little studied. In 1958, Ralph Lewin emphasized the importance of macroalgal genetics (Lewin, 1958); now, more than 50 years later, still very few mutants have been isolated and analyzed. In addition to the difficulties of growing macroalgae in laboratory conditions and their complicated and relatively long life cycles, there is a surprising lack of interest for this field of study. As a result—and despite the amenability of haploid organisms for genetic and molecular analyses of underlying mechanisms and pathways—the identification of genes involved in the control of marine macroalgal growth and development lags far behind that of other studied multicellular organisms (Maluszynski et al., 1995; Howell, 1998).

The most advanced genetic characterization of developmental mutants in a multicellular alga has been carried out in the Chlorophyceae taxon *Volvox carteri* (sub-division Chlorophyta; Leliaert et al., 2012). This microscopic freshwater alga is composed of about 2000 bi-flagellated cells stuck together within a thick extracellular gelatinous matrix, forming a moving spherical body. While most cells remain flagellated and vegetative, 16 cells differentiate into non-flagellated, larger asexual reproductive cells (gonidia) from asymmetric cell divisions (Starr, 1969). Subsequent developmental steps lead to embryo inversion, externalizing the flagellated somatic cells and internalizing the gonidia.

Chemical mutagenesis and transposon-tagging in *V. carteri* produced several morphologically impaired mutants (Sessoms and Huskey, 1973). The *glsA* gene, coding for a chaperone protein involved in both protein translation and transcriptional regulation (Miller and Kirk, 1999; Pappas and Miller, 2009), controls the asymmetric cell division giving rise to the gonidia cells. Additional mutants led to the identification of transcription factors controlling genes specific to either the gonidial cells (*lag* gene) or the somatic cells (*regA* gene) (Kirk, 2003). In addition, the *invB* mutant, impaired in the inversion process, has been shown to code for a nucleotide-sugar transporter (Ueki and Nishii, 2009) necessary for the expansion of the glycoprotein-rich gonidia vesicle, which tightly surrounds the multicellular sphere before inversion.

The use of temperature-sensitive transposons to generate both tagged mutants and revertants, and the use of genetic transformation to complement the mutants and further analyze them at the molecular level are important assets of *Volvox* genetics because they allow rapid molecular identification of the causal genes (Ueki and Nishii, 2008). A similar approach can be used in macroscopic and marine macroalgae. To date, only a very few morphological mutants have been characterized—primarily at the phenotypical level. In compensation for their complex life cycles and sometimes challenging culture conditions, these mutants often display similar or increased reproductive capabilities in lab conditions compared to the wild types (*Ulva mutabilis*, Fjeld and Løvlie, 1976). Mutants were obtained either from sampling natural stocks, or among offspring of mutagenized populations.

#### Naturally-induced mutations in marine macroalgae

**Figure 3:**
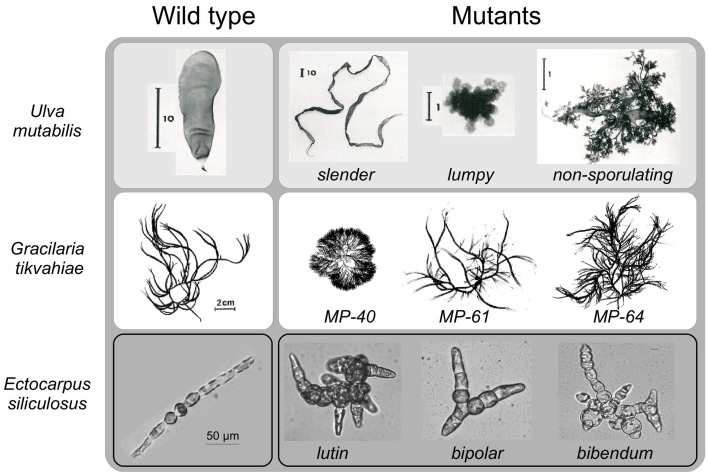
**Morphology of some marine macroalgal mutants**. Examples of some morphological mutants of the green macroalga *Ulva mutabilis*, the red macroalga *Gracilaria tikvahiae* at mature stages, and the brown macroalga *Ectocarpus siliculosus* at early stages. Unless otherwise specified, the scale bar is the same as for the wild-type individual. Permission to reproduce the images of *Ulva mutabilis* (Fjeld and Løvlie, 1976) and *Gracilaria tikvahiae* (Patwary and van der Meer, 1982) was granted by John Wiley & Sons, Inc. and the journal *Botany*, respectively. *Ectocarpus*: personal photos (B. Charrier).

Some macroalgal lines are particularly prone to spontaneous mutations. The resulting phenotypes often revert to the wild type, indicating that the mutations are unstable, with most being attributed to a particularly unstable locus. However, there have been some stable mutants that have lent themselves to genetic analyses (Föyn, 1961). One laboratory strain of the green marine macroalga *U. mutabilis* has been shown to be particularly inclined to spontaneous morphological changes with a rate 10 times higher than other strains (Fjeld and Løvlie, 1976). Some generated mutants displaying growth and morphological features different from the wild type parent have been reported (Föyn, 1959). The *slender* mutant (Figure 3) displays a higher growth rate and impairment in cell differentiation and cell enlargement, because only small and undifferentiated cells are observed (Løvlie, 1969). Similarly, cells show altered polarized division and cell differentiation in the *lumpy* mutant, which develops as a loose aggregate of undifferentiated cells with no specific organs (Bryhni, 1973, Figure 3). In this mutant, the composition of the cell wall is modified, with an increased proportion of water-extractable polysaccharides, resulting in higher plasticity of the cell wall (Bryhni, 1978). It is not clear whether the inhibition of cell differentiation is a result of increased cell wall plasticity. In contrast, the *globose* mutant is small and seems to have a more active differentiation process, developing many more rhizoids and a darker thallus than the wild type (Föyn, 1961, not shown). There are other distinct phenotypes, less well described (e.g., non-sporulating mutant, Figure 3). Løvlie (1978) also showed developmental variations in *U. mutabilis* temperature-sensitive mutants. The mutants develop normal phenotypes, forming a filament consisting of a row of cells at 22°C, whereas at 15°C they produce abnormal phenotypes.

In the brown alga *Ectocarpus siliculosus*, the natural *immediate upright* mutant shows a flaw in the early morphogenesis of the filamentous sporophyte, in which the prostrate basal body is reduced, thereby resembling a gametophyte with mainly upright filaments (Peters et al., 2008). This phenotype is due to a single-locus mutation, emerging in the offspring population (gametophytes) from a wild-type sporophyte, probably by spontaneous random mutation.

#### Chemically or UV-light induced mutations in marine macroalgae

Various mutagenic agents have been used in marine macroalgae (Figure 1E). Chemical agents, such as N-methyl-N'-nitro-N-nitrosoguanidine and ethyl-methane sulfonate (EMS) are efficient. EMS has been used to produce red macroalga *G. tikvahiae* mutants (van der Meer, 1979), which display altered branching patterns and thallus thickness, potentially due to the modification of the size of the medullary and sub-cortical cells of the algal thallus (Patwary and van der Meer, 1982). These morphological alterations are accompanied by a modification in the agar composition: agar strength is higher in the MP-40 mutant, but lower in the MP-61 mutant (Patwary and van der Meer, 1983a) (Figure 3). Interesting biological issues therefore lie in the potential functional link between the biophysical characteristics of agar and the morphological alterations observed in these mutants. The demonstration of this functional link requires adequate segregation analyses of these characters in the progeny of the mutants, which, unfortunately, were not carried out at that time. Nevertheless, and of interest for aquaculture purposes, the MP-40 mutant displays a faster growth rate than the wild-type even at high densities. It also fixes ammonium better from seawater and is more resistant to epiphytic colonization (Patwary and van der Meer, 1983b). In contrast to most generated mutations that were single locus and recessive, the MP-40 mutant displayed a partly dominant mutation (Patwary and van der Meer, 1982). Unavailable in the 1980s, modern technologies can now use this genetic feature to identify the mutated gene.

The use of chemical mutagens in liquid media represents a major drawback due to the risk of contamination during waste elimination. UV irradiation is safer and as efficient, and has been used on the green macroalga *U. mutabilis* and the brown macroalga *E. siliculosus*. Half-life curves related to the mutagen dose have been established (Le Bail and Charrier, 2013). Exposition of gametes to UV-irradiation doses corresponding to 50% survival produce only single-locus mutations in *Ectocarpus* (*étoile* mutant, Le Bail et al., 2011, *orobourous* mutant, Coelho et al., 2011; *mut* mutant, Billoud et al., 2015), making them amenable to relatively simple genetic analyses. A library of 60 morphological mutants has been constructed for *E. siliculosus*, which all show a stable phenotype after five rounds of sub-culturing or five parthenogenetic generations (Le Bail and Charrier, 2013). The observed morphological defaults cover all the developmental steps in this alga, ranging from alterations in cell differentiation and filament growth polarity, to positional and stage modification in the branching process (Figure 3). The transition to the reproductive phase is altered in some mutants. Interestingly, most mutants are affected in several of these developmental features, indicating that the function of the wild-type gene is not constrained to a single, precise developmental stage, but spans different developmental phases. This is consistent with the low level of architectural complexity of the *Ectocarpus* body, which is mainly composed of filaments (Le Bail et al., 2008).

Because these mutations were generated randomly and were not tagged, their identification requires forward genetic approaches and molecular tools, unavailable at the time. Today however, the advent of cost-effective and improved molecular and sequencing techniques can help tackle the genetic characterization of these mutants, and even identify the causal mutation, and the gene directly responsible for the morphological alteration. The recently described next-generation sequencing-based mapping approach developed for *E. siliculosus* (Billoud et al., 2015) should pave the way to the other macroalgal mutants generated from seaweeds with preferably small genome sequences.

## Conclusion and future prospects

Evidence that intensive cultivation of inbred seaweed lines results in both an impoverishment of wild biodiversity (possibly by gene swamping) and an outbreak of infections and epiphytism in culture plants (Loureiro et al., 2015), has raised awareness in aquaculture stakeholders and policy-makers that the inventory, the maintenance and the exploitation of seaweed genetic resources should be fully deployed (Pullin, 2006; Pullin and White, 2011; FAO, 2013). Hence, initiation and development of long-term seaweed genetic improvement programs are recommended, which include continuous selection processes targeting the maintenance of genetic diversity throughout the production of improved lines (Robinson et al., 2013). Macroalgal stocks with altered morphological traits can either be continuously collected from the wild or generated on demand in culture facilities using a combination of techniques likely to modify the developmental traits in a stable way. Although somatic hybridization frequently gives unpredictable results, generating hybrids between macroalgal partners displaying interesting and stable morphological features—obtained for example by induced mutagenesis—can combine both traits in a single organism. Using this approach, Mizukami et al. (1995) generated hybrids between a wild type and a mutant strain of *P. yezoensis*.

In addition to the interest of these techniques for aquaculture, production of morphologically altered macroalgae is a very valuable tool for studying the genetics of basic developmental mechanisms. In comparison with the wild-type organism, a mutant can provide information about cell types, specific developmental steps and gene expression patterns that play a role in the impaired developmental process, and may lead to the identification of the causal gene itself. In this regard, the development of genetic studies should be undertaken to address some biological issues that can only otherwise just be touched upon. Genetic transformation, of little use in the aquaculture sector (Robinson et al., 2013), can target specific cellular mechanisms by selectively modifying the expression of key genes, as it has been performed for decades in land plants. Genetic transformation of macroalgae is now possible in red and green seaweeds (Mikami, 2014; Wichard et al., 2015) and should be further developed, especially in brown algae, so that genetics and transgenesis can be used to their full potential in the field of macroalgal developmental biology.

Altogether, the current stage of technological knowledge now allows access to new and more powerful ways of generating morphologically altered macroalgae, using both traditional methods, such as the production of somaclonal variants (whose phenotype cannot be predicted and controlled), and newly developed gene-targeted methods, such as creating macroalgal variants with specific, precise and predictable phenotypes. The combined use of these methods will help bring the field of macroalgal development up to speed, to match that of land plants.

### Conflict of interest statement

The authors declare that the research was conducted in the absence of any commercial or financial relationships that could be construed as a potential conflict of interest.
