# Supplementary material for: Production of genetically and developmentally modified seaweeds: exploiting the potential of artificial selection techniques
Source: Front Plant Sci. 2015 Mar 17;6:127. doi: 10.3389/fpls.2015.00127 (PMC4362299; doi:10.3389/fpls.2015.00127)
Supplement: Supplementary file 1 [file Table1.DOC]

### Supplementary Table 1| List of seaweeds being used as food in different countries (Source: Zemke-White and Ohno 1999).

| **Seaweed Species** | **Country** |
| --- | --- |
| *Capspsiphon fulvescens* | Korea |
| *Caulerpa* spp | Malaysia, Thailand |
| *Caulerpa lentillifera* | Philippines |
| *Caulerpa peltata* | Philippines |
| *Caulerpa racemosa* | Bangladesh, Japan, Philippines, South Pacific Islands, Vietnam |
| *Caulerpa sertularioides* | Philippines |
| *Caulerpa taxifolia* | Philippines |
| *Codium* spp | Argentina |
| *Codium bartletti* | Philippines |
| *Codium edule* | Philippines |
| *Codium fragile* | Korea, Philippines |
| *Codium muelleri* | Hawaii |
| *Codium taylori* | Israel |
| *Codium tenue* | Indonesia |
| *Codium tomentosum* | Indonesia |
| *Colpomenia sinuosa* | Philippines |
| *Enteromorpha* spp | Bangladesh, France, Hawaii, Myanmar |
| *Enteromorpha compressa* | Korea, Indonesia |
| *Enteromorpha clathrata* | Korea |
| *Enteromorpha grevillei* | Korea |
| *Enteromorpha intestinalis* | Indonesia, Japan, Korea |
| *Enteromorpha linza* | Korea |
| *Enteromorpha nitidum* | Korea |
| *Enteromorpha prolifera* | Indonesia, Japan, Korea, Philippines |
| *Monostroma nitidum* | Japan |
| *Scytosiphon lomentaria* | Korea, France |
| *Ulva* spp. | Argentina, Canada, Chile, Hawaii, Japan, Malaysia |
| *Ulva lactuca* | Vietnam Indonesia |
| *Ulva reticulata* | Vietnam |
| Acanthophora spicifera | Philippines, Vietnam |
| Asparagopsis taxiformis | Hawaii, Indonesia |
| Betaphycus gelatinum | Vietnam |
| Calaglossa adnata | Indonesia |
| Catenella spp | Myanmar |
| Chondria crassicaulis | Korea |
| Chondrus crispus | Ireland, France |
| Chondrus ocellatus | Japan |
| Eucheuma cartilagineum | Japan |
| Eucheuma gelatinae | Indonesia, Japan, Philippines, Caribbean |
| Eucheuma muricatum | Indonesia |
| Gelidiella acerosa | Philippines |
| Gelidiella tenuissima | Bangladesh |
| Gelidium spp | Hawaii |
| Gelidium anansii | Korea, Indonesia |
| Gelidium latifolium | Indonesia |
| Gelidium pusillum | Bangladesh |
| Gloiopeltis spp | Vietnam |
| Gracilaria changii | Thailand |
|  |  |
| Table 1 continues |  |
| Seaweed Species | **Country** |
| Gracilaria cornea | Caribbean |
| Gracilaria coronopifera | Hawaii, Vietnam |
| Gracilaria crassissima | Caribbean |
| Gracilaria domingensis | Brazil, Caribbean, Chile |
| Gracilaria eucheumoides | Indonesia, Vietnam |
| Gracilaria firma | Vietnam |
| Gracilaria fisheri | Thailand |
| Gracilaria gracilis | Vietnam |
| Gracilaria lemaneiformis | Japan |
| Gracilaria parvispora | Hawaii |
| Gracilaria salicornia | Thailand, Vietnam |
| Gracilaria tenuistipitata var. liui. | Thailand, Vietnam |
| Gracilaria verrucosa | France, Indonesia, Japan, Korea |
| Grateloupia filicina | Indonesia, Japan |
| Halymenia spp. | Myanmar |
| Halymenia discoidea | Bangladesh |
| Halymenia durvillaei | Philippines |
| Hypnea spp | Myanmar |
| Hypnea muscoides | Vietnam |
| Hypnea nidifica | Hawaii |
| Hypnea pannosa | Bangladesh, Philippines |
| Hypnea valentiae | Vietnam |
| Iridaea edulis | Iceland |
| Kappaphycus alvarezii | Philippines |
| Kappaphycus cottonii | Vietnam |
| Laurencia obtusa | Indonesia |
| Laurencia pinnitifida | Portugal |
| Mastocarpus stellatus | Ireland |
| Mazzaella splendens | Canada |
| Meristotheca papulosa | Japan |
| Meristotheca procumbens | South Pacific Islands |
| Nemalion vericulare | Korea |
| Palmaria hecatensis | Canada |
| Palmaria mollis | Canada |
| Palmaria palmata | Canada, France, Iceland Ireland, UK, US |
| Porphyra spp | Israel, New Zealand, UK |
| Porphyra abbottae | Alaska, Canada |
| Porphyra acanthophora | Brazil |
| Porphyra atropurpurae | Indonesia |
| Porphyra columbina | Argentina, Chile, Peru |
| Porphyra crispata | Thailand, Vietnam |
| Porphyra fallax | Canada |
| Porphyra haitanensis | China |
| Porphyra kuniedae | Korea |
| Porphyra leucostica | Portugal |
| Porphyra perforata | Canada |
| Porphyra psuedolanceolata | Canada |
| Porphyra seriata | Korea |
| Porphyra spiralis | Brazil |
| Porphyra suborbiculata | Korea, Vietnam |
| Porphyra tenera | Japan, Korea |
| Porphyra torta | Alaska, Canada |
| Porphyra umbilicalis | France, US |
|  |  |
|  |  |
|  |  |
| Table 1 continues |  |
| Seaweed Species | **Country** |
| Porphyra vietnamensis | Thailand |
| Porphyra yezoensis | China, Japan, Korea |
| Pterocladia capillacea | Korea |
| Scinaia moniliformis | Philippines |
| Solieria spp | Myanmar |
| Alaria crassifolia | Japan |
| Alaria fitulosa | Alaska |
| Alaria marginata | Canada |
| Alaria esculenta | Iceland, Ireland, US |
| Cladosiphon okamuranus | Japan |
| Cladosiphon okamuranus | Japan |
| Durvillaea antarctica | Chile |
| Ecklonia cava | Japan |
| Ecklonia stolonifera | Korea |
| Egregia menziesii | Canada |
| Fucus serratus | France |
| Fucus vesiculosus | France, Portugal |
| Hizikia fusiformis | Japan, Korea |
| Hydroclathrus clathratus | Bangladesh, Philippines |
| Laminaria angustata | Japan |
| Laminaria bongardiana | RoK Alaska |
| Laminaria diabolica | Japan |
| Laminaria digitata | Ireland |
| Laminaria groenlandica | Canada |
| Laminaria japonica | China, Japan, Korea |
| Laminaria longicruris | US |
| Laminaria longissima | Japan |
| Laminaria octotensis | Japan |
| Laminaria religiosa | Japan, Korea |
| Laminaria saccharina | Alaska, Canada, Ireland, Rok Alaska |
| Laminaria setchelli | Canada |
| Macrocystis pyrifera | Argentina |
| Nemacystis decipiens | Japan |
| Nereocystis luetkaena | US |
| Pelvetia siliquosa | Korea |
| Postelsia spp | US |
| Sargassum aquifolium | Indonesia |
| Sargassum crassifolium | Thailand |
| Sargassum spp. | Bangladesh, Hawaii, Malaysia, Myanmar, Philippines, Thailand, Vietnam |
| Sargassum filipendula | Egypt |
| Sargassum horneri | Korea |
| Sargassum oligosystum | Thailand |
| Sargassum polycystum | Indonesia, Thailand |
| Sargassum siliquosum | Indonesia |
| Undaria pinnitifida | Australia, China, Japan, Korea |
| Undaria peterseniana | Korea |
